# Development of Poly(acrylamide)-Based Hydrogel Composites with Powdered Activated Carbon for Controlled Sorption of PFOA and PFOS in Aqueous Systems

**DOI:** 10.3390/polym15224384

**Published:** 2023-11-11

**Authors:** Maria Victoria X. Klaus, Angela M. Gutierrez, J. Zach Hilt

**Affiliations:** 1 Department of Chemical and Materials Engineering, University of Kentucky, Lexington, KY 40506, USA; mxklaus@uky.edu (M.V.X.K.); amgu232@g.uky.edu (A.M.G.); 2 Superfund Research Center, University of Kentucky, Lexington, KY 40506, USA

**Keywords:** hydrogels, PFAS, PAC

## Abstract

Per- and polyfluoroalkyl substances (PFAS) are anthropogenic compounds developed for various applications; some are connected to adverse health impacts including immunosuppression and higher susceptibility to some cancers. Current PFAS remediation treatments from aqueous sources include granular activated carbon (GAC) adsorption, membrane separation, and anion-exchange resin (AER) removal. Each has specific disadvantages, hence the need for a new and efficient technology. Herein, acrylamide-based hydrogel composites were synthesized with powdered activated carbon (PAC) and characterized to determine their affinity for PFAS. Physicochemical characterization included Fourier-Transform infrared spectroscopy (FTIR) to identify chemical composition, thermogravimetric analysis (TGA) to confirm PAC loading percentage, and aqueous swelling studies to measure the effect of crosslinking density. FTIR showed successful conversion of carbonyl and amine groups, and TGA analysis confirmed the presence of PAC within the network. Surface characterization also confirmed carbon-rich areas within composite networks, and the swelling ratio decreased with increasing crosslinking density. Finally, sorption of PFAS was detected via liquid chromatography with tandem mass spectrometry (LC-MS/MS), with removal efficiencies of up to 98% for perfluorooctanoic sulfonic acid (PFOS) and 96% for perfluorooctanoic acid (PFOA). The developed hydrogel composites exhibited great potential as advanced materials with tunable levers that can increase affinity towards specific compounds in water.

## 1. Introduction

For decades, per- and polyfluoroalkyl substances (PFAS) have been manufactured for a plenitude of products due to their repellent nature and have, since, persisted in nature in large concentrations causing worldwide concern [1]. The industrialization of PFAS has plagued the environment and our drinking water; therefore, exposure to humans and animals is inevitable [2]. Perfluorooctanoic acid (PFOA) and perfluorooctanesulfonic acid (PFOS) are two of the most prominent ”legacy” PFAS compounds. With both, there is sufficient evidence of association to several detrimental effects on human health such as dyslipidemia, decreased response to antibodies, and increased risk of kidney cancer [3,4]. In 2022, the United States Environmental Protection Agency (EPA) set a new 0.004- and 0.02-parts-per-trillion (ppt) interim health advisory level (HAL) for PFOA and PFOS in drinking water, respectively (compared to the previous HAL of 70 ppt for the pair), but levels beyond parts-per-billion (ppb) have been reported in various aqueous sources as well as the human body [5,6]. Current treatments for PFAS remediation in aqueous systems include granular activated carbon (GAC) adsorption, reverse osmosis and nanofiltration membrane separation, and anion-exchange resin (AER) removal [7,8,9]. Adsorption by GAC relies on hydrophobic interactions facilitated by a large surface area that can capture upwards of 90% of PFAS in certain cases, but has shown unselective behavior when competing species are present [10]. Membrane separation is based on its semi-permeable nature through size exclusion and on the manipulation of chemical properties through functionalization to increase affinity towards PFAS; however, materials that do not pass through the membrane can accumulate if they are unable to be removed from the surface, which leads to fouling and loss of efficiency [11]. The electrostatic interaction between certain negatively charged PFAS molecules and the cationic AERs results in ionic bonds, and the polymeric resin produces Van der Waals forces with the hydrophobic tail of the contaminants [7,11]. The performance of AERs in removing PFAS is dependent on several factors including polymer matrix and functional groups; they have also proven costly [7]. In conclusion, the drawbacks of these methods are significant; hence, the need for a new cost-effective and efficient technology for PFAS removal is of utmost importance [7,8,9,10].

Novel materials based on natural and synthetic polymer sorbents have recently gained attention for remediation of PFAS in aqueous systems due to their high water-retention capacity and low production cost [10,12]. In particular, hydrogels, i.e., crosslinked hydrophilic polymers that swell in aqueous systems whilst maintaining their physical and chemical integrity, have been investigated [13]. These materials can be easily functionalized with co-monomers and/or hydrophobic crosslinkers that enhance their sorptive properties [14]. Furthermore, hydrogel composites are polymer networks containing embedded particulates that can also introduce hydrophobic interactions and are of high interest for environmental applications [15,16]. Among natural compounds, cellulose is the most abundant polymer in nature and can be extracted into its microcrystalline form and functionalized with poly(ethylenimine) (PEI) for increased sorption of the negatively charged PFAS head. Ateia et al. demonstrated that the adsorption capacity of cellulose for PFOA and PFOS at the initial capacity of 1 μg/L was within 70–80% removal in the first 100 s [17]. Moreover, researchers have explored the incorporation of ionic fluorogels, through thermally initiated radical copolymerization of perfluoropolyethers (PFPE), with methacrylate chain-end functionality (Fluorolink MD 700) and an amine-containing monomer (2-dimethylaminoethyl methacrylate, DMAEMA) for further affinity for PFAS. A variety of both short- and long-chain compounds, including PFOA and PFOS, were removed from wastewater treatment plant-collected sample water with efficiencies of >95% [18]. Furthermore, chitosan, an amine-containing polysaccharide, has also exhibited adsorptive properties towards these contaminants due to the ionic interactions between the carboxylic/sulfonic groups of PFAS and the cationic groups of the polymer [19]. Affinity for specific contaminants can be enhanced by crosslinking with epoxy epichlorohydrin (ECH) in the presence of the contaminant, forming a molecularly imprinted hydrogel that still adsorbs over 50% of PFAS in the presence of competing pollutants [20]. Ateia et al. have reported on the development of poly(N-[3-(dimethylamino)propyl] acrylamide, methyl chloride quaternary) (DMAPAA-Q) hydrogels for PFAS sequestration [12]. Again, both short- and long-chain compounds were analyzed in a range of pH solutions with removal efficiencies beyond 80%, and full removal of select sulfonic contaminants was also achieved. Although novel adsorbent materials are promising platforms for PFAS removal from water, uncertainty regarding potential toxic effects from the bare adsorbents and the possible limitations of scalability have hindered research in this area; therefore, they need to be further explored [21].

**Figure 1:**
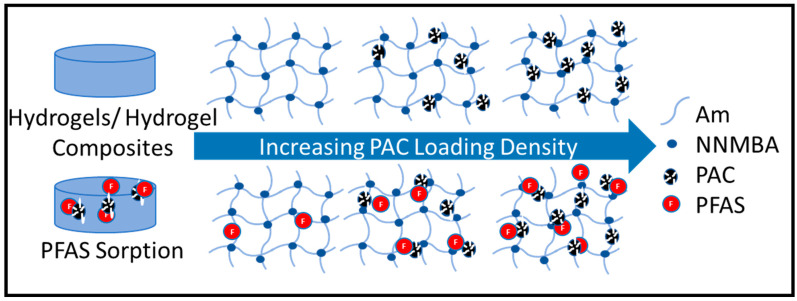
Visual innovation of materials which show an increase in PFAS removal efficiency with increasing PAC loading density.

Even though GAC is well-known for its sorptive properties and the binding capacity of PFAS, powdered activated carbon (PAC) exhibits higher binding capacity due to its greater surface area, shorter internal diffusion, better site accessibility, and faster adsorption kinetics than GAC. Son et al. calculated the removal efficiency of PAC from various sources and activation methods by removing nine PFAS compounds, which exhibited removal exceeding 80% of most carbons for five analytes, including PFOA and PFOS, and at least 10% for short-chain compounds [22]. Nonetheless, the smaller diameter size (normally below 0.1 mm) of the powdered activated carbon limited these materials from being implemented in large-scale columns as separation and regeneration have proven inefficient [7]. Researchers have developed a stable aqueous suspension of PAC within a poly(diallyldimethylammonium chloride) (DADMAC) network to remove PFAS from water systems, with adsorption capacities of 379.8 mg/g and 495.2 mg/g for PFOA and PFOS, respectively [23]. The combination of a hydrophobic material within a hydrogel network allows for improved contact between sorbent and contaminants in aqueous systems [16]. Herein, we report on the robust synthesis of a poly(acrylamide) (PAm)-powdered activated carbon hydrogel composite with an affinity for PFAS. A series of crosslinking densities were explored to understand the impacts on polymer properties; increasing PAC loading percentages was also explored to measure the effects of hydrophobic interactions, as shown in Figure 1. Physicochemical characterization of the materials was performed using infrared spectroscopy and thermogravimetric analysis to determine conversion of the chemical bonds of monomer to polymer and PAC loading percentage, respectively. Surface characterization was also analyzed for most systems using high imaging microscopy and energy-dispersive X-ray spectroscopy. In addition, the swelling capacity of the composites and the removal efficiency of two PFAS contaminants (PFOS and PFOA) were also studied.

## 2. Materials and Methods

### 2.1. Materials

Acrylamide (Am) (CAS No. 79-06-1), N,N-methylenebisacrylamide (NNMBA) (CAS No. 110-26-9), sodium hydroxide (NaOH) (CAS No. 1310-73-2), and ammonium persulfate (APS) (CAS No. 7727-54-0) were purchased from Sigma-Aldrich (Milwaukee, WI, USA). Tetramethylethylenediamine (TEMED) (CAS No. 110-18-9) was received from TCI Chemicals (Tokyo, Japan). Carbon powder, activated, Norit GSX, steam-activated, acid-washed (CAS No. 7440-44-0) was purchased from Alfa Aesar, with a surface area of 694.7 ± 93.9 nm (determined using dynamic light scattering). Finally, PFAS such as PFOA (95% purity) (CAS No. 335-67-1) and PFOS (97% purity) (CAS No. 1763-23-1) were received from Alfa Aesar and Strem Chemicals (Milwaukee, WI, USA), respectively. All the chemical components were used as received. To construct a synthesis reaction cell, a pair of microscope glass plates, a polytetrafluoroethylene (PTFE) spacer, and binder clips were used.

### 2.2. Preparation and Physicochemical Characterization of Hydrogels and Hydrogel Composites

To synthesize the hydrogel, a solution was prepared by mixing the acrylamide monomer, the chemical crosslinker, NNMBA and, when applicable, the PAC particulate, together in a 20 mL borosilicate scintillation vial using a 10 mol basis of feed in a 1:2 ratio of deionized (DI) water. By varying the feed ratio of the monomer to the crosslinker and PAC loading percentage, a series of hydrogels and hydrogel composites were synthesized to investigate the size-restriction capability of the network and PAC effects. The feeding compositions of the prepared gel samples are summarized in Table 1. The resulting solutions were thoroughly mixed using a vortex, and the hydrogel composites were additionally subjected to probe sonication using a Model 500 Sonic Dismembrator (Fisher Scientific, Hampton, VA, USA) for increased dispersion of PAC for 1 min with 5 s on pulses and 10 s off in between. To initiate the free-radical polymerization reaction, a redox system consisting of APS and TEMED was applied. A 10 wt% APS solution in deionized (DI) water at concentrations of 0.2, 0.8, and 1 wt% of the total feed mass for the neat (0% PAC), 1% PAC, and 5% PAC systems, respectively, was added simultaneously with 1/5 of the APS volume of TEMED. The polymerization was carried out at 25 °C. This exothermic reaction can be altered by controlling the amount of the initiator/catalyst composition, which can increase the kinetics for the composites as the reaction needs to occur faster to keep the particles from settling. For the neat acrylamide systems, reaction occurred within 30 min while, for the composites, the reaction occurred within 2 min. The solution was then immediately transferred into the reaction cell, where a PTFE frame of 1.6 mm thickness was sandwiched between a pair of parallel microscope glass slides with a disposable pipette.

**Figure 2:**
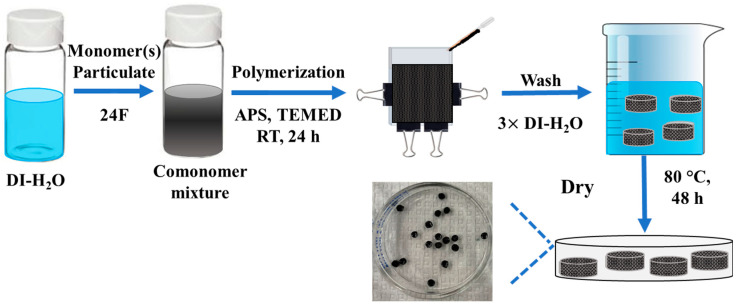
Schematic of hydrogel and hydrogel composite synthesis.

Following polymerization, the hydrogel films were then removed from the reaction cell mold and subsequently immersed in DI water to remove any unreacted chemical components and for spontaneous hydration. After 24 h, the materials were then punched into circular disks using a 6 mm diameter metal cutter, and the water was changed. The water was replaced one more time, after 48 h, to obtain the satisfactory removal of any unreacted monomers and initiators from the solution. Finally, the swollen polymers were dried at 80 °C for 48 h and stored in a scintillation vial until further use as shown in Figure 2.

Fourier transform infrared (FTIR) spectra were obtained on a 7000e FTIR spectrometer, with attenuated total reflectance sampling technology (Varian Inc., Palo Alto, CA, USA). For analyzing polymers, a fine powder is desired for direct contact with the crystal; this was achieved here using a pestle and mortar. The scanning range was 700–4000 cm^−1^ and the resolution was 8 cm^−1^. Thermogravimetric analysis (TGA) was performed on a Q600 SDT dynamic scattering calorimetry (DSC)-TGA (TA Instruments, New Castle, DE, USA), under nitrogen gas from 20 °C to 110 °C at 20 °C/min; The temperature remained isothermal for 15 min to evaporate any free water or physically bound molecules. After the water was removed, the temperature was then ramped at 10 °C/min to reach 800 °C and held isothermal for 15 min before the completion of the experiment. TGA was used to determine the incorporation of PAC loading percentage of hydrogels and hydrogel composites. The amount of PAC that was determined to be incorporated into the polymeric network can be calculated by the following equation, Equation (1):

(1) PACcompositefinalwt%−neathydrogelfinalwt%=PACloading%

### 2.3. Mass Yield of Monomer and Crosslinker to Polymer Conversion

Mass yield was investigated to determine how much of the monomer/crosslinker converted into a final polymeric material by comparing the feed mass of Am and NNMBA with the final mass of dry polymers. The mass yield was calculated by using Equation (2):

(2) $$\left(\frac{\mathrm{final}\mathrm{dry}\mathrm{mass}}{\mathrm{initial}\mathrm{dry}\mathrm{mass}}\right)\times 100\%=\%\mathrm{Mass}\mathrm{Yield}$$

Initial dry mass was calculated by summing the feed components with the exception of the solvent (DI water).

### 2.4. Surface Characterization of Sorbent Materials

Surface characterization of hydrogels and hydrogel composites was conducted using a Quanta FEG-250 scanning electron microscope (SEM) (FEI, Lausanne, Switzerland), for high-resolution surface imaging and by analyzing the composition by energy-dispersive X-ray microanalysis (EDS). SEM and EDS were conducted for 1NNT, 5NNT, 10NNT, 10N1P, and 10N5P materials. Samples were prepared by placing materials on conductive carbon tape and sputter coating with 5 nm of platinum utilizing an EM ACE600 sputter coater (Leica, Wetzlar, Germany).

### 2.5. Aqueous Swelling Experiments of Hydrogels and Hydrogel Composites

Swelling behavior indicates the expansibility of the materials in water; this was studied under environmental conditions following a common gravimetric method. The dry hydrogel circular disks were immersed into a 1M sodium hydroxide-titrated deionized water solution (pH = 7.0 ± 0.2) at 25 °C. During the swelling process, the samples (*n* = 3) were taken out of the solution at scheduled time points, blotted with Kimwipe tissues to remove excess surface water, weighed, and re-immersed into the water. These steps were repeated at the desired time point(s) to obtain the complete kinetic swelling profile. For kinetic studies, samples were massed at 1, 4, 8, 12, and 24 h; equilibrium studies were performed at 4 h, which were determined by the kinetic experiments. The mass swelling ratio (q) was calculated by using Equation (3). To calculate the equilibrium mass swelling ratio of composites, the following equation was used:

(3) $$q=\frac{Ws}{Wd}$$

where Ws and Wd represent the weights of the swollen gel and the dry gel, respectively.

### 2.6. PFAS Sorption Studies

The sorption capability of the materials to bind PFAS was determined by an individual batch experiment. A mixture of PFOA and PFOS at initial concentrations of 100 μg/L each, with an adsorbent concentration of 0.5 mg/mL, was exposed to 30 mL of 1 M NaOH-titrated deionized water solution (pH = 7.0 ± 0.2) in 50 mL centrifuge tubes at 25 °C. All hydrogels and hydrogel composites were assessed, as well as controls comprised of solvent only, neat hydrogels and solvent, and PAC only. The mixtures were shaken at room temperature (25 °C) on a temperature-controlled orbit shaker at 200 rpm. After 24 h of interaction, samples were collected and filtered using a Whatman 4 filter with 90mm diameter; PFOA and PFOS were analyzed via a Vanquish Core high-performance liquid chromatography (HPLC) system coupled with a TSQ Altis Plus Triple Quadrupole Mass Spectrometer (MS) (Thermo Fisher Scientific, Waltham, MA, USA). The mobile phase consisted of (A) LCMS-grade water and (B) LCMS-grade methanol: water (85:15), both with 20 mM ammonium acetate (Sigma Aldrich). The column was heated and held constant at 40 °C. The LCMS was operated in negative polarity mode with electrospray ionization. The limit of detection (LOD) for target analytes was 1 ng/L; seven calibration points with linear dynamic range (LDR) were within 1–150 μg/L and had R^2^ values of 0.9989 and 0.9981 for PFOA and PFOS, respectively. Calibration standards and instrument blanks were run at the beginning and end of the analytical run. Batch experiments were performed in triplicates (*n* = 3) and the results have been reported as an average ± standard deviation. The removal efficiency was calculated using Equation (4) as follows:

(4) $$RE=\frac{\left(Co-Ct\right)}{Co}*100\%$$

where *Co* and *Ct* are the initial and residual concentrations of PFAS analytes in (μg/L), respectively.

## 3. Results and Discussion

Acrylamide-based hydrogel and hydrogel composites were successfully synthesized using free-radical polymerization to obtain a series of materials with varying crosslinking densities and PAC loading percentages to determine impacts on physicochemical properties. The sorbent systems were also characterized and analyzed for their binding affinity to PFAS.

### 3.1. Synthesis Confirmation and Physicochemical Characterizations

**Figure 3:**
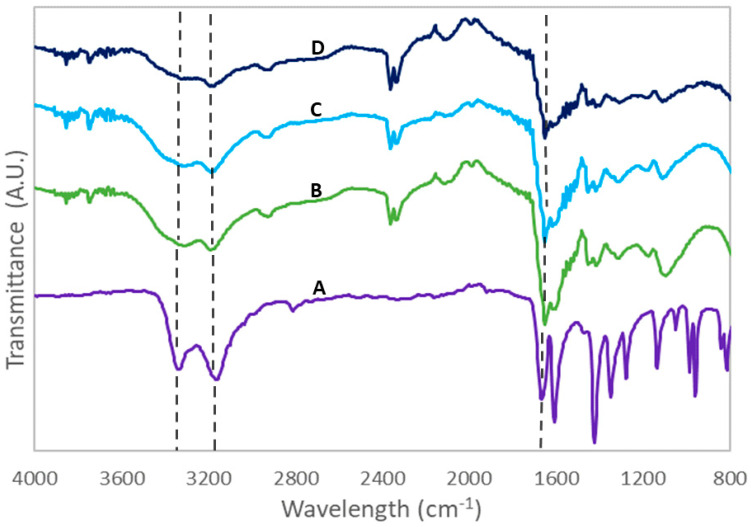
FTIR spectra performed with ATR technologies of (A) plain acrylamide, and hydrogels and hydrogel composites with increasing PAC loading density, (B) 5NNT hydrogels, (C) 5N1P composites, and (D) 5N5P hydrogels, respectively.

Mass conversion was calculated for all systems, with >90% yield for both neat and 1% PAC hydrogels and hydrogel composites, whereas the 5% PAC loaded systems drastically decreased in yield, as shown in Table 1. This indicates a disruption of the polymerization reaction with increasing particulate concentration, which could be attributed to the adsorption of the initiator/catalyst (APS and TEMED) by the PAC during the reaction. Additionally, since the PAC is a solid particulate that is physically entrapped within the hydrogel network, it can impact the network formation during polymerization and consequently impact yield. As shown in Figure 3 and Figure S1, the IR spectra of all PAm systems confirmed successful polymerization of the monomer and crosslinker into polyacrylamide through the absorption of expected peaks. Characteristic carbonyl and amide functionalities were indicated by the absorption peaks between 1640 and 1660 cm^−1^ corresponding to the dashed line in between those peaks which revealed the formation of the primary bending amide (NH_2_) band and the stretching carbonyl (C=O) group. The doublet peaks between 3100 and 3400 cm^−1^ further account for amide functionality through the stretching of both primary (NH2) and secondary (NH) groups as highlighted by the two dashed lines within the corresponding peaks [24,25,26]. Additionally, the absence of the alkene peak (C=C) around 1610 cm^−1^ indicates the high conversion of the acrylamide monomer to polymer [25]. Figure 3 elucidates the impacts of PAC loading density by comparing 5% crosslinked hydrogel composites, with 1 and 5% PAC, with the 5NNT system. All characteristic peaks, i.e., carbonyl peaks between 1640 and 1660 cm^−1^ and amide peaks between 3100 and 3400 cm^−1^, reduced in intensity as the amount of PAC was increased, which can again be attributed to the adsorption of APS and TEMED by PAC and the subsequent inhibition of polymerization. In contrast, FTIR spectra were also analyzed to compare the influence of varying crosslinking densities between the three neat systems (1, 5, and 10%), as displayed in Figure S1. No significant trends were observed between the three differing crosslinking concentrations within diagnostic peaks.

**Figure 4:**
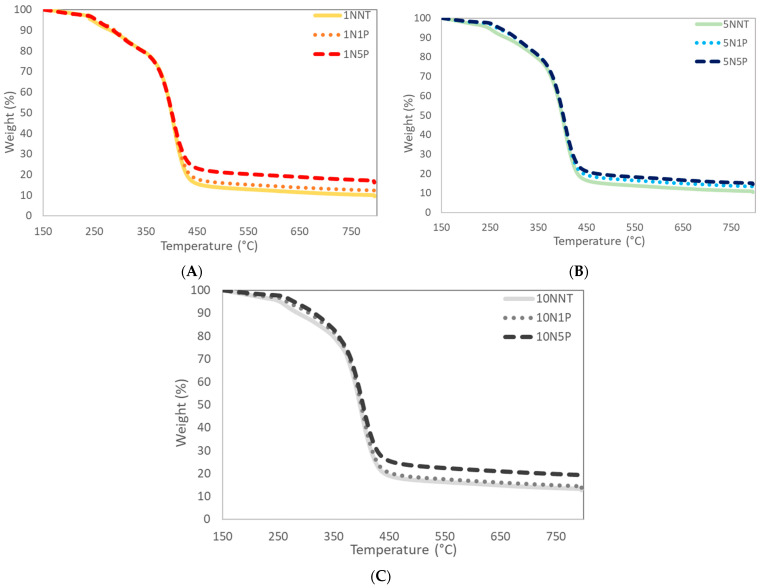
TGA analyses of hydrogels and hydrogel composites in Nitrogen gas from 150 °C to 800 °C to determine PAC loading percentages in (**A**) 1% crosslinked systems, (**B**) 5% crosslinked hydrogels, and (**C**) 10% crosslinked polymers, all with varying PAC loading percentages.

The TGA curves for the hydrogels and hydrogel composites were normalized to weight at 150 °C to account for water evaporation in order to obtain the hydrogels’ and composites’ degradation as shown in Figure 4. The normalized graphs shown in Figure 4 display a two-stage decomposition, where the first mass loss, from 250 to 375 °C, is assigned to the loss of ammonia as a result of the imidization reaction of the amide groups, which accounted for roughly a 20% weight decrease; the second sharp mass loss zone, from 375 to 450 °C, indicates the breakdown of imide groups formed in the first stage and polymer backbone by the liberation of H_2_, CO, and NH_3_, resulting in the final carbonaceous residues [12,25,27]. The residual amount of carbon stabilized out at roughly 10% of the initial weight, and this amount was used as the baseline to determine actual PAC loading percentage. After normalizing the neat weight (%) that remained following degradation, the percentage of PAC was determined for the 1% and 5% loaded systems seen in Table 1. Both the 5% and 10% crosslinked systems had PAC loading percentages within 1.13% max deviation from the expected values of 1 and 5 initial PAC loading percentages, whereas the 1% crosslinked systems had a higher deviation comparatively (upwards of 2.5% for the system with 5% PAC), which is consistent with the assumption that the particulate PAC is disrupting the polymerization reaction. Since the 1% systems have fewer crosslinked networks and less conversion of polymeric material, the loading percentage appears higher given that there is less polymeric feed.

### 3.2. Surface Characterization

**Figure 5:**
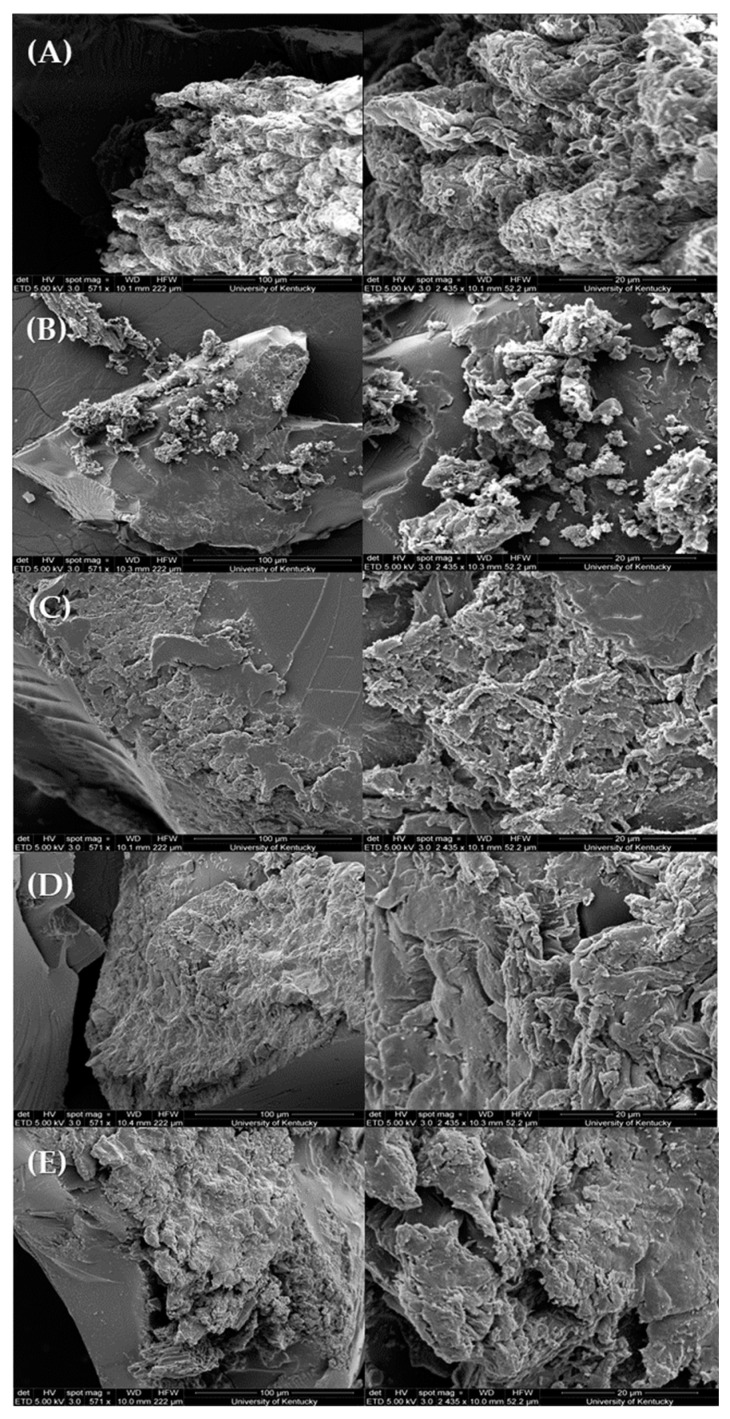
Scanning electron microscopy images of neat hydrogels with (**A**) 1%, (**B**) 5%, and (**C**) 10% NNMBA to measure impacts of increasing crosslinking density, and (**D**) 10N1P and (**E**) 5% PAC composites to determine the effect of PAC inclusion within the network with scale bars of 100 μm on the left, at lower magnification, and 20 μm on the panels on the right.

SEM and EDS were analyzed for 1NNT, 5NNT, 10NNT, 10N1P, and 10N5P materials to determine any impacts on morphology due to varying crosslinking densities (1, 5, and 10%) and PAC loading percentages (0, 1, and 5%). High-resolution images for all systems are shown in Figure 5. Figure 5A–C compare the morphology of the three neat materials with different crosslinking densities, i.e., 1NNT, 5NNT, and 10NNT, at two magnifications; no significant differences in morphology were observed. Additionally, Figure 5C–E compare how the varying PAC loading concentrations impact morphology; again, there were no significant differences in morphology noted; however, EDS microanalysis of 10% crosslinked systems with and without PAC indicates the inclusion of carbon-rich spots with increasing PAC loading percentages, represented by the concentrated blue marks on the EDS maps of composites in Figure S2.

### 3.3. Aqueous Swelling Studies

#### 3.3.1. Kinetic Swelling Studies

**Figure 6:**
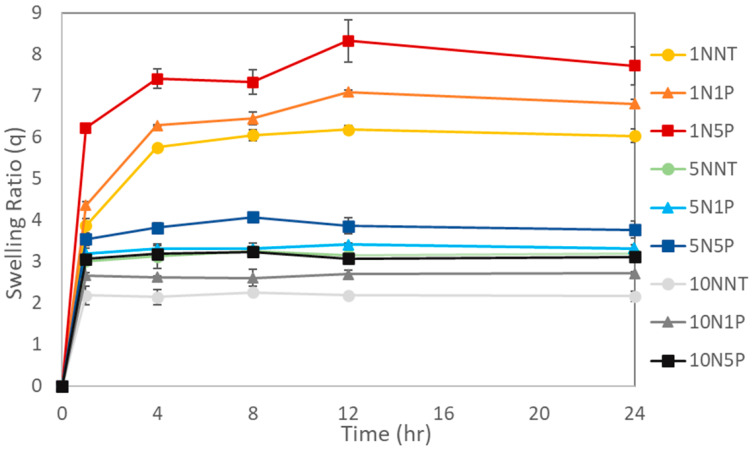
Kinetic swelling behavior of hydrogels and hydrogel composites in DI water after 1, 4, 8, 12, and 24 h.

The swelling kinetics of the hydrogels and hydrogel composites containing various NNMBA and PAC contents at 1, 4, 8, 12, and 24 h are displayed in Figure 6. For all cases, the swelling profiles exhibited rapid swelling with equilibrium swelling reached by approximately 4 h. The initial swelling was dominated by the capillary adsorption interaction from the porous structure of the hydrogels and hydrogel composites. The second phase was attributed to the saturation of the polymeric network by free diffusion of the water molecules until the swelling equilibrium was reached [28].

#### 3.3.2. Equilibrium Swelling Studies

**Figure 7:**
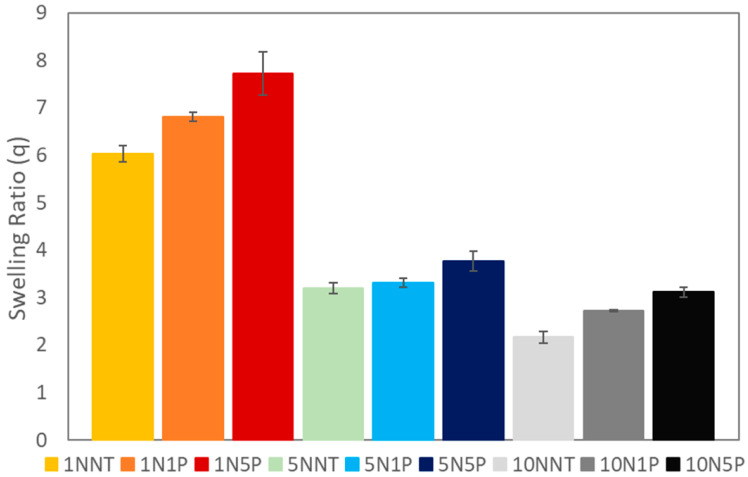
Aqueous equilibrium swelling behavior of all hydrogels and hydrogel composites in DI water after 24 h.

Swelling behavior measures the expansibility of materials and determines water-retention properties. A high swelling ratio indicates a large amount of water that the network can absorb and a more open network, which can impact the ability of molecules of different sizes to absorb into the system. As expected, hydrogels with increased NNMBA contents exhibited a decrease in water uptake due to a higher crosslinking density and a more restricted network, as illustrated in Figure 7. Moreover, there was an increase in equilibrium swelling with increasing PAC loading percentage, which can be attributed to a decrease in polymer conversion due to the physical inhibition of the polymerization reaction by the particulate PAC addition. The hydrogels experienced swelling ratios in the range of 2–8 where the least adsorption was displayed as 2.16 ± 0.12 for the neat hydrogel with the highest crosslinking density (10N), and the highest swelling ratio was displayed by the least crosslinked system (1N) with the largest PAC loading (5P), with 7.73 ± 0.46.

### 3.4. Equilibrium Sorption Studies

**Figure 8:**
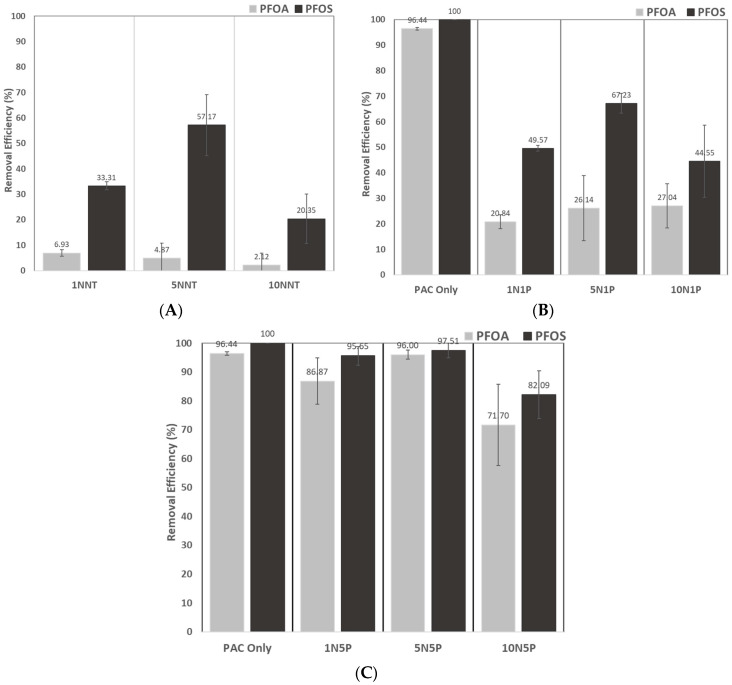
PFOA and PFOS removal efficiency of sorbent materials of PFAS at a starting concentration of 100 μg/mL in a 12 mL DI-water solution titrated to obtain a pH 7 with NaOH and 0.5 mg/mL. A PAC-only control was also tested and is compared against (**A**) the neat hydrogels, (**B**) PAC composites with 1% PAC loading, and (**C**) 5% PAC systems.

The removal efficiency of the hydrogels and hydrogel composites for PFAS was determined for all systems using a mixture of PFOA and PFOS at an initial concentration of 100 ppb of each, as shown in Figure 8. No sorption was observed for the systems with no sorbent, demonstrating that the filters are not absorbing any contaminant and, therefore, are not interfering with the reported results. Neat hydrogels revealed limited sorption ability with a maximum binding capacity of PFAS analytes with a maximum of 57% removal for PFOS for the 5% crosslinked system and 6.93% PFOA from the 1NNT system, with no significant trends between varying crosslinking densities, as seen in Figure 8A. The sorption mechanism of PFAS binding to the neat hydrogels is likely attributable to the physisorption of the PFAS analytes through Van der Waals electrostatic interactions [29,30]. The addition of PAC within the hydrogel network showed significantly higher binding percentages than those seen for the neat hydrogels visible in Figure 8B,C. PFAS removal by composites with 1% PAC increased by >10% for all three crosslinking densities, with the 5% crosslinking density exhibiting a slightly higher removal efficiency between all of the three polymers for PFOS. Hydrogel composites with the highest PAC loading percentages (5% PAC) exhibited removal efficiencies upwards of 72% for PFOA and 82% for PFOS, with a maximum PFOA and PFOS removal of 96% and 98%, respectively, by the 5N5P system. Although there was not a significant trend between the 1 and 5% crosslinked systems, the 10N5P sorbent showed the potential restriction of these longer-chain PFAS due to the increase in crosslinking density (i.e., higher NNMBA concentration). The PAC-only systems showed virtually 100% removal efficiency for both PFOS and PFOA. The improved binding of the PAC systems is explained by the expected hydrophobic interactions of the PFAS tail and activated carbon surface. All composite systems exhibited higher removal of PFOS compared to PFOA, which is consistent with the literature, which has found that the removal of long-chain and sulfonic PFAS is dominated by hydrophobic interactions [12]. Moreover, the sorption studies were performed in NaOH-titrated DI-water solution, which introduced Na^+^ ions following dissolution and increased the ionic charge, which enhanced PFOS binding and had little effect on PFOA sorption [31].

Although PAC outperformed all sorbents in removing PFOA and PFOS, it has proved to be highly unselective towards short-chain compounds when competing with legacy analytes as its major pores are saturated with legacy compounds and therefore block the binding of short-chain contaminants. It was hypothesized that modifying the NNMBA concentration of the material and increasing its crosslinking density would limit the removal percentage of larger PFAS molecules due to a more restrictive network and, therefore, allow for more sorption of short-chain compounds. The binding data suggest that this hypothesis could be accurate, given that the systems with the highest crosslinking densities appear to limit some of the PFOA and PFOS sorption. This hypothesis will be tested further with follow-up studies that explore additional crosslinking densities and binding studies, including competing short-chain PFAS. The findings from this study indicate the high sorption potential of PFAS contaminants and introduce an exciting platform that can be further modified for increased selectivity of contaminants, such as a composite with added cationic functionality.

## 4. Conclusions

Overall, acrylamide-based hydrogel and hydrogel composite sorbents were successfully synthesized using free-radical polymerization and characterized using several methods including mass yield, FTIR, and TGA. Mass yield was the primary determining factor for polymer conversion by making sure the feed materials were transferring to the final product. Congruently, FTIR confirmed the presence of the carbonyl and amide peaks within the polymeric systems that were also present in the analogous monomer. TGA analysis further indicates polymeric degradation between 300 and 450 °C, which is consistent with hydrogel breakdown, and the residual weight observed at 800 °C confirmed the incorporation of PAC within the hydrogel composites at each respective loading capacity, which increased with increased loading percentage. SEM analysis concluded that there were no significant changes between the images with varying crosslinking densities but confirms the inclusion of PAC within hydrogel composites by carbon-rich spots in EDS maps. Kinetic swelling studies were performed to determine the time required for the systems to reach equilibrium, which was shown to be reached within 4 h for all systems. This swelling time was then used to calculate equilibrium swelling ratios for all systems, where the highest swelling ratio was attributed to that with the least crosslinked network, as expected, with a decrease as the crosslinking density was increased. The addition of PAC particulate also enhanced the swelling capacity of the hydrogels, with the 5% PAC loaded systems having a higher swelling ratio than the 1% PAC, both being higher than the neat systems.

Finally, neat hydrogels showed limited sorption of the PFAS analytes with only 50% capacity for the 5NNT systems, whereas the composites showed greater sorption of both PFOA and PFOS. The higher sorption of PFOS above PFOA was attributed to the electronegativity of the sulfonic group of PFOS being higher than the carboxylic group in PFOA. The findings from this study indicate the high sorption potential of PFAS contaminants in these hydrogel composites, and these materials can be further modified for increased selectivity and investigated for a wider range of analytes.

## Figures and Tables

**Table 1:** Feed compositions of hydrogels and hydrogel composites with mass yield percentage ranging from 69% to 99% and varying PAC final loading percentages.

Hydrogel/ Hydrogel Composite Samples	Crosslinking Density Am:NNMBA (mol%)	PAC Loading Percentage (mass %)	Mass Yield %	PAC Final Loading %
1NNT	99:1	-	91	-
5NNT	95:5	-	98	-
10NNT	90:10	-	99	-
1N1P	99:1	1	90	2.44
5N1P	95:5	1	95	1.49
10N1P	90:10	1	96	1.00
1N5P	99:1	5	78	7.50
5N5P	95:5	5	69	3.87
10N5P	90:10	5	79	5.89

## Data Availability

The data presented in this study are available on request from the corresponding authors.

## Supplementary Materials

The following supporting information can be downloaded at: https://www.mdpi.com/article/10.3390/polym15224384/s1, Figure S1: FTIR spectra of neat hydrogels with increasing crosslinking density as follows, (a) 1NNT, (b) 5NNT, and (c) 10NNT. Figure S2: Scanning electron microscopy energy-dispersive X-ray microanalysis maps of 10% crosslinked systems with (a) no activated carbon, (b) 1% PAC, and (c) 5% PAC.
