# Supplementary material for: Development of Poly(acrylamide)-Based Hydrogel Composites with Powdered Activated Carbon for Controlled Sorption of PFOA and PFOS in Aqueous Systems
Source: Polymers (Basel). 2023 Nov 11;15(22):4384. doi: 10.3390/polym15224384 (PMC10675425; doi:10.3390/polym15224384)
Supplement: Supplementary file 1 [file polymers-15-04384-s001.zip › polymers-2692751-supplementary.pdf]

Supplementary Material

Development of poly(acrylamide)-based hydrogels and hydrogel composites with PAC for controlled sorption of PFOA and PFOS in aqueous systems

M. V. X. Klaus[a,b], A. M. Gutierrez[a,b] , and J. Z. Hilt[a,b,*]

a. Department of Chemical and Materials Engineering, University of Kentucky, 177 F Paul Anderson Tower, Lexington, KY 40506, USA

b. Superfund Research Center University of Kentucky Lexington, KY 40506, USA

*Corresponding author.

Email-address: hilt@uky.edu

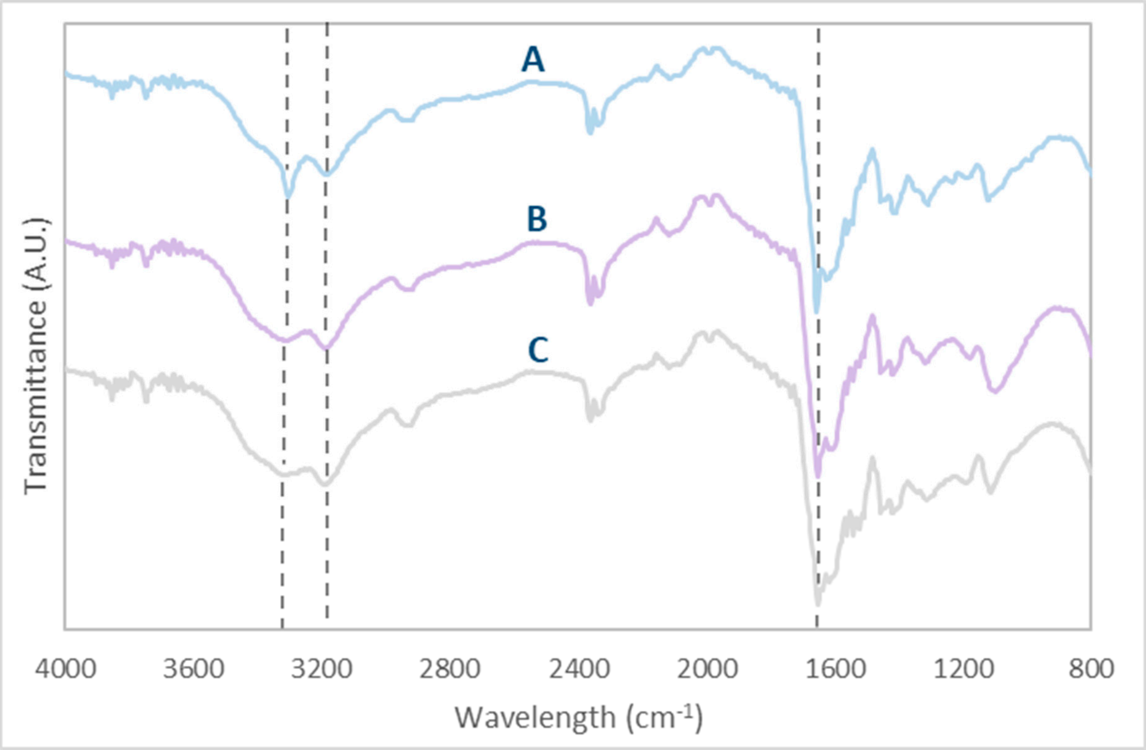

Figure S1 FTIR spectra of neat hydrogels with increasing crosslinking density as follows, A) 1NNT, B) 5NNT, and C) 10NNT.

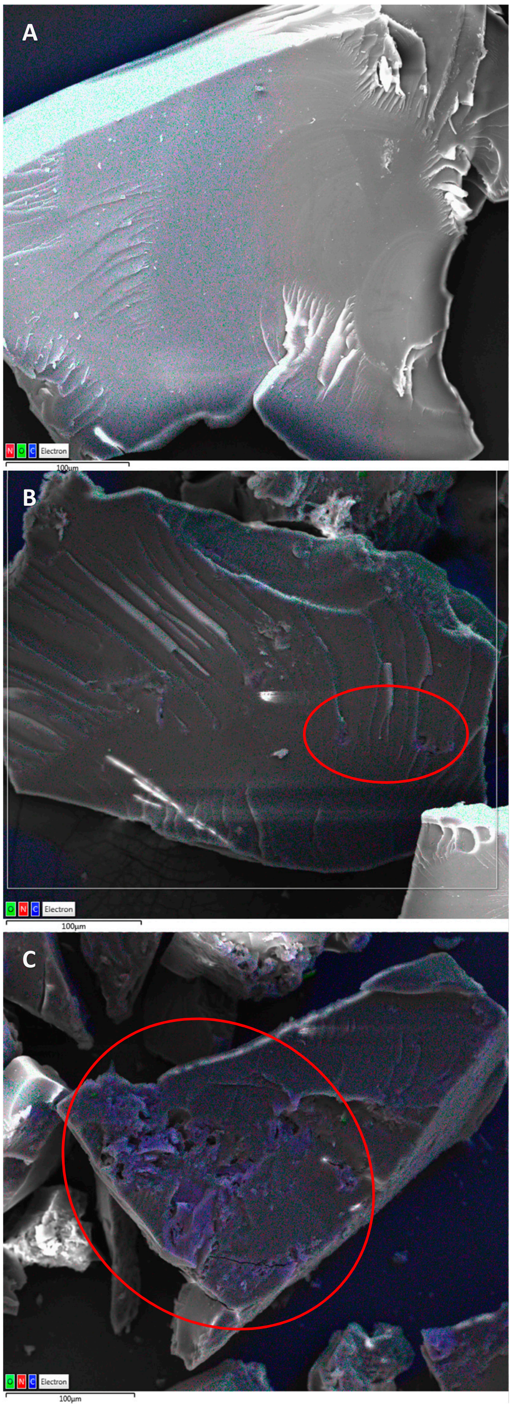

Figure S2 *Scanning electron microscopy energy-dispersive X-ray microanalysis maps of 10% crosslinked systems with A) no activated carbon, B) 1% PAC, and C) 5% PAC*
